# The logic of monsters: development and morphological diversity in stem-cell-based embryo models

**DOI:** 10.1098/rsfs.2024.0023

**Published:** 2024-10-25

**Authors:** Dominica Cao, Sumit Garai, James DiFrisco, Jesse V. Veenvliet

**Affiliations:** ^1^ Department of Molecular, Cellular, and Developmental Biology, Yale University, New Haven, CT 06520, USA; ^2^ Theoretical Biology Lab, The Francis Crick Institute, London NW1 1AT, UK; ^3^ Division of Biosciences, Medical Sciences Building, University College London, Gower Street, London WC1E 6BT, UK; ^4^ Stembryogenesis Lab, Max Planck Institute of Molecular Cell Biology and Genetics, Dresden 01307, Germany; ^5^ Center for Systems Biology Dresden, Dresden 01307, Germany; ^6^ Cluster of Excellence Physics of Life, Technische Universität Dresden, Dresden 01307, Germany

**Keywords:** stem-cell-based embryo models, gastruloids, Pere Alberch, developmental constraints, evo-devo, teratology, morphospace, organoids

## Abstract

Organoids and stem-cell-based embryo models (SEMs) are imperfect organ or embryo representations that explore a much larger space of possible forms, or morphospace, compared to their *in vivo* counterparts. Here, we discuss SEM biology in light of seminal work by Pere Alberch, a leading figure in early evo-devo, interpreting SEMs as developmental ‘monstrosities’ in the Alberchian sense. Alberch suggested that ordered patterns in aberrant development—i.e. ‘the logic of monsters’—reveal developmental constraints on possible morphologies. In the same vein, we detail how SEMs have begun to shed light on structural features of normal development, such as developmental variability, the relative importance of internal versus external constraints, boundary conditions and design principles governing robustness and canalization. We argue that SEMs represent a powerful experimental tool to explore and expand developmental morphospace and propose that the ‘monstrosity’ of SEMs can be leveraged to uncover the ‘hidden’ rules and developmental constraints that robustly shape and pattern the embryo.

## 1. Introduction

“‘The greatest illusion,’ said the mole, ‘is that life should be perfect’”.

—Charlie Mackesy

Empirical biology studies actual diversity, with possible but unrealized forms being extrapolated and inferred from actual organisms. Experimental and historical limits on this process mean that we typically do not know how big the space of possible forms, or ‘morphospace’ [1–3], is. This is true both for whole organisms and for traits like the tetrapod limb, somitogenesis or avian gastrulation. Experiments in molecular developmental biology traditionally target individual genes under controlled conditions, but combining a series of narrow, gene-centric perturbation experiments does not necessarily yield a global understanding of complex developmental processes, which often entail feedback between genetic and non-genetic information modules (biochemistry, mechanics and geometry) [4–7]. There is a wide gap between the kind of experimental understanding we have and what would be needed to answer broader questions about morphospace, such as—what other tetrapodal limb morphologies could have evolved in the ancestral sarcopterygian?

Today, the new technology of stem-cell-based embryo models (SEMs) is enabling the experimental exploration of developmental morphospaces to an unprecedented degree. The techniques involve coaxing pluripotent stem cells (PSCs) to form aggregates that, under defined regimes of culture conditions, can self-organize into structures that recapitulate aspects of natural embryos. Being simplified representations of their *in vivo* counterparts, SEMs typically lack many of the constraints and redundancies of natural development. Hence, SEMs can be conceptualized as the result of large-scale, constructive disruptions that can effectively nudge a developmental system to develop into different regions of morphospace. Although far from providing anything like an exhaustive probe of morphospace even for a single developmental process, SEMs can nonetheless reveal otherwise hidden morphogenetic possibilities. They are thereby delivering insight into normal development—for example, on the relationship between gene expression and cell and tissue mechanics, self-organization, the role of extracellular matrix (ECM) and extra-embryonic tissues and more.

In this paper, we explore the significance of SEM biology in light of Pere Alberch’s (1989) work on developmental ‘monstrosities’ [4]. A leading early figure in evo-devo, Alberch argued that monstrosities—dramatic pathological morphologies resulting from abnormal development—could uniquely contribute to our understanding of development and evolution, and specifically to understanding why morphospace is ‘partially filled’. This is because ordered patterns of monstrosities—e.g. the fact that two-headed vertebrates and cyclopia are relatively common whereas three-headed vertebrates and three-eyed mammals are not—indicate the presence of developmental constraints on possible forms, constraints not due to natural selection. We suggest that SEMs are ‘monsters’ in the Alberchian sense, despite only occurring in the laboratory. After providing an overview of Alberch’s (1989) ‘logic of monsters’ [4], we describe some of the ways in which current thinking departs from his views of developmental constraints, selection and evolution. We suggest that, although monstrosities may not be as directly consequential for evolution as he thought, they are highly consequential for development for the basic reason that pathology provides insight into the structure of normal conditions. We describe the unique opportunities offered by SEMs to obtain novel insights into developmental morphospaces.

Box 1. **Glossary**

**Stem-cell-based embryo model (SEM):** multicellular aggregates formed *in vitro* by subjecting PSCs to various signals, allowing them to self-organize, grow and differentiate into structures reminiscent of natural embryos or aspects thereof. SEMs include models such as gastruloids and blastoids but not organoids or two-dimensional (2D) cultures.

**Morphospace:** the space of possible forms or morphologies, as defined by a set of parameters or axes, with filled regions corresponding to actually existing morphologies. A morphospace may be depicted more narrowly or broadly, e.g. for a given trait, organism or clade. For a useful discussion of properties of different types of formal morphospaces, see Mitteroecker & Huttegger [3].

**Monster:** an organism with dramatically pathological or extreme morphology due to abnormal development, such as a two-headed vertebrate.

**Developmental constraint:** a concept that can be differentiated into mechanistic and variational meanings, which are not mutually exclusive. In the *mechanistic* sense, a developmental constraint is a structure, condition, mechanism or process that ‘constrains’ or influences another developmental process in the same organism by reducing its degrees of freedom and that typically exists at a higher spatiotemporal scale than the constrained process. In the *variational* sense, a developmental constraint is a limit on the production of variation in a population due to aspects or mechanisms of development. This is a contested concept (see §2.3).

**Internal selection:** differential survival or reproduction in a population caused by interaction between traits within the organism, as opposed to interaction between trait and environment.

**Stabilizing selection:** selection in which the trait optimum is a particular intermediate (non-extreme) value relative to the population trait distribution, which tends to ‘stabilize’ the population trait mean at that value. This is contrasted with directional selection, in which the trait optimum is an extreme value and which tends to move the trait mean in the direction of that extreme.

**Robustness:** the stability of a phenotypic attribute to genetic change (genetic robustness) or environmental change (environmental robustness) or change in conditions generally. Stability or robustness of outcome to change of conditions is synonymous with *canalization*.

**Plasticity:** the propensity of a phenotype to take on different states (e.g. bright or dark colouration) without change of genotype, usually due to interaction with the environment or with other traits in the organism.

**Self-organization:** a process in which nonlinear interactions between system components lead to emergent order or patterns at a higher size scale than the components, without significant external instructive guidance.

**Elongation:** the process whereby an embryo extends its primary axis in an anteroposterior direction.

**Somitogenesis:** the process of generating somites, segmented blocks of mesodermal tissue, which eventually develop into various structures such as the dermis of the skin, skeletal muscles and bones of the spine.

**Geometry:** the shape, size and relative arrangement of the parts of a system.

**Boundary condition:** mathematically, boundary conditions are conditions that the solution(s) of a differential equation must satisfy at the boundary of the space on which solutions are defined, e.g. by taking specified values at the boundary. This is contrasted with initial conditions, which are conditions the solution(s) of the equation must satisfy at a specified start time *t*_0_. Informally and in the context of morphogenesis, boundary conditions are mechanistic *constraints* located typically at the physical boundaries of a tissue or an embryo.

## 2. Revisiting Alberch’s ‘logic of monsters’: developmental constraints in morphospace

Pere Alberch was an important early contributor to evolutionary–developmental biology (see [8–10]), especially to the core theoretical proposal that development influences evolution by structuring the variation upon which selection acts [11]. The last decades have seen empirical progress in explaining evolutionary patterns in terms of developmental processes. At the same time, we have also seen conceptual progress in understanding some of the central notions Alberch invoked, such as developmental constraints, selection and morphospace. Below, we introduce the main idea of Alberch’s seminal 1989 paper ‘The logic of monsters’ [4], while also detailing differences from current thinking, in order to set up its contemporary relevance for interpreting SEMs.

### 2.1. Evolutionary internalism

Alberch’s (1989) paper [4] is ostensibly a historical review of ‘teratology’, the science of ‘monstrosities’. Monstrosities are dramatic pathological morphologies resulting from aberrant development, such as two-headed vertebrates. The interest in developmental monstrosities is motivated by the fact that they can be interpreted, as the paper’s subtitle expresses, as ‘Evidence for internal constraint in development and evolution’. Alberch noted that pathological morphologies are not random or continuously distributed in morphospace (i.e. the space of possible morphologies, see Glossary), but exhibit ‘order’ and discreteness. He assumed monstrosities to be lethal and eliminated by selection, unlike the ‘hopeful monsters’ idea of Goldschmidt [12], which suggested that large-scale mutational changes were responsible for saltational evolution. Alberch reasoned that because developmental monstrosities exhibit ordered patterns of diversity, and because these patterns could not have evolved by stepwise selection over many generations, their ‘orderliness’ must be explained by internal constraints on the generative rules of development. The fact that two-headed vertebrates sometimes naturally occur but three-headed vertebrates seemingly never occur must be explained by mechanisms of axial and cranial morphogenesis in vertebrates rather than by adaptation to the environment. More generally, Alberch suggests, developmental morphospaces are only partially filled, and much of the patterns of actual diversity we see (e.g. in tetrapod limbs [13]) are due to constraints on the generative rules of development rather than natural selection.

**Figure 1.**
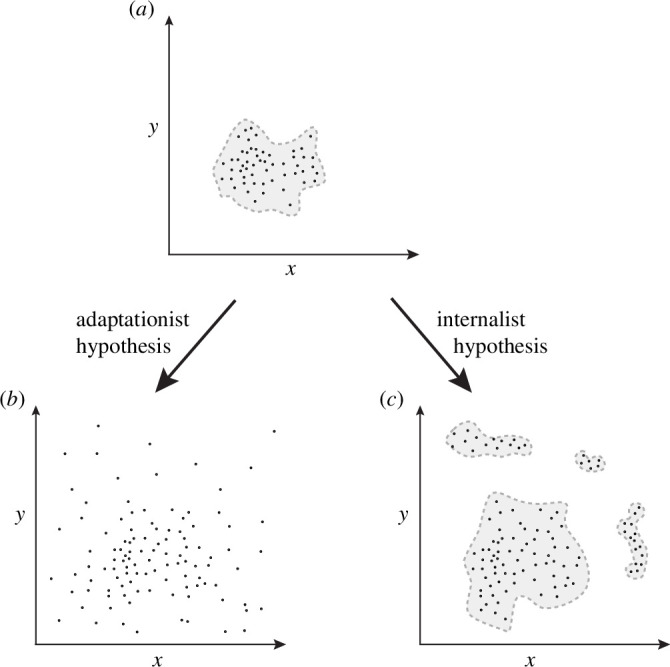
Alberch’s ‘internalist’ argument as visually illustrated in a figure, redrawn after Alberch [20]; cf. Amundson [21]. If we take an existing form such as the forewing of *Drosophila melanogaster* and plot it in a 2D morphospace (*a*), defined by parameters such as proximodistal length versus anteroposterior length, the natural species variation would occupy a subregion of that space. Why is this? The adaptationist answer is that unoccupied regions represent forms that have been eliminated by selection. This reasoning leads to the following hypothetical experiment: if we were to somehow remove or minimize the effects of natural selection while maintaining reproduction, the population variation should diffuse outwardly in an unconstrained fashion (*b*). By contrast, the internalist view holds that partial occupancy of morphospace is due to developmental constraints. Removing the effect of natural selection might change the shapes and distribution of occupied regions, but morphospace would remain only partially filled (*c*). These clusters can be viewed as polyphenisms or as forms in different species (e.g. wings of different Drosophilds). Developmental monstrosities putatively represent a natural experiment of this sort (without reproduction), one that according to Alberch fits the internalist hypothesis (*c*).

Alberch classifies his argument as a form of evolutionary *internalism*, more often known as ‘structuralism’ [14–18]. We can define internalism as follows. Evolution is a two-part process comprising (i) the *generation* of genotypic and phenotypic variation by processes such as mutation, recombination, and development and (ii) the *sorting* of variation by population-level processes such as natural selection, migration and drift. *Internalism* is the view that generative processes (i) are more important for explaining evolutionary patterns and outcomes. *Externalism*, on the other hand, is the contrary view that the sorting process (ii) is more important for explaining evolutionary patterns and outcomes, with ‘adaptationism’ being the predominant externalist view emphasizing the sorting of variation by selection [19]. As Alberch [4, p. 27] writes, ‘Internalist theory argues that even in the absence of selection naturally occurring morphologies would be clumped and gaps would be prominent in the distribution of morphologies’ (see figure 1). Monstrosities exhibit these gaps due to *developmental constraints*, Alberch argues, but the same is true of normal tetrapod limbs, which exhibit stereotyped development and limited morphological diversity [13]. In contrast, the externalist explanation for the morphological stasis of tetrapod limbs is, he suggests, that they share a common ancestor, and it has not been adaptively necessary to change their basic developmental design, which he deems insufficient as an explanation [4].

The first difficulty with the above-mentioned argument for internalism is that any evolutionary pattern, such as the distribution of biological diversity in morphospace, will be a result of *both* generative (i) and sorting (ii) processes working together in long-term feedback. If internalism and externalism are competing causal hypotheses, it is very difficult to test them in normal population settings by holding either process constant while letting the other process vary. Even if a teratological organism cannot reproduce, for example, it is the product of developmental systems with a history of natural selection. This history no doubt influences which teratologies are accessible from existing populations, phenotypes and genomes. This is—at least to some extent—also true for SEMs since the starting material (PSCs) has a history of natural selection (discussed in §4.2).

In assessing the question of whether generative or sorting processes are more important, a general answer has been that it depends on the nature of the variation. Gould [22] hypothesized that selection would be more important for explaining an evolutionary pattern or outcome if the available genetic and phenotypic variation is abundant, small and occurring in any direction with equal likelihood, i.e. ‘isotropic’ [23]. Generative processes, in contrast, would be more important when the available genetic and phenotypic variation is more limited and ‘anisotropic’. Though theoretically plausible, in most practical contexts the required information about the nature of available variation will be too limited to discriminate between these alternatives.

### 2.2. Internalism versus internal selection

A more fundamental difficulty with the internalist argument of ‘The logic of monsters’ is that the internal–external distinction does not neatly map onto the development–selection distinction. Alberch formulates a description of evolution that initially seems similar to the one provided above: ‘Order in evolution is a combination of deterministic agents at two levels: internally generated order based on the internal dynamics of development, and natural selection, dependent on the properties of organism–environment interaction’ [4, p. 46]. Yet natural selection is not necessarily a matter of organism–environment interaction. More abstractly, natural selection occurs when there are trait differences in a population that cause differential survival or reproduction. Adaptive response to selection requires these trait differences to be heritable. Trait differences can cause differential survival or reproduction due to interaction with the environment (e.g. camouflage and pathogen defence), or alternatively due to interaction with other traits in the organism. In the process of *internal selection* [24–26], selection occurs on a trait due to its developmental or physiological relationship to other traits in the same organism, a relationship that may hold across many or all environments. Many of the clearest examples of internal selection involve *stabilizing selection* against disruptive changes in development, which may result in embryonic lethality. For example, in vertebrates, Sonic Hedgehog (Shh) secreted from the notochord is necessary for induction of the floor plate and ventral patterning of the neural tube and somites [27,28]. Individuals in a vertebrate population that possess variations in this signalling system that are insufficient for neural patterning (e.g. Shh mutants) do not survive to maturity, very likely in all environments. Notochord signalling is most likely maintained at appropriate levels in vertebrate populations by internal selection.

This is relevant for understanding SEMs because they can still be subjected to a form of internal selection despite their highly modified laboratory environment. Although all SEMs are ultimately non-viable and cannot reproduce, they can exhibit variation in the capacity to progress to certain stages corresponding to stages of natural embryo development. To this extent, they constitute populations with differential viability, and this may be used to model internal selection in natural embryos. As a potential example of internal viability selection in SEMs, trunk-like structures (TLS) establish somites and a neural tube in the absence of a notochord, but the resulting dorsalization of neural tube patterning [29] might impinge on their capacity to transition to further differentiated states, ultimately limiting their viability.

There is evidence suggesting that many non-molecular traits experience stabilizing selection [30–34]. Once we recognize the existence of internal selection and the pervasiveness of stabilizing selection, knowing what would happen to a developmental system ‘in the absence of selection’ becomes challenging. Selection does not only act after development has been completed. It acts throughout the entire life cycle [35], with both internal and external selection on viability occurring throughout embryonic development. Patterns of diversity (‘order’) in embryonic development therefore are not necessarily indicative of internal constraints on development *in the absence* of selection, since they will already be biased by viability selection in the embryo. The ‘monsters’ that have been historically recorded are those with enough developmental viability that they can grow to a detectable size, be born or hatch and be observed. There may be developmental systems in ‘unoccupied’ regions of morphospace that get selected-out before this can occur.

**Figure 2.**
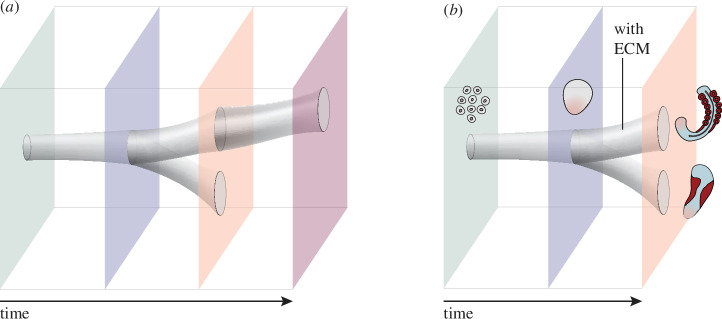
(*a*) A time-extended morphospace can capture the fact that some ontogenies are only viable up to stages prior to completed morphogenesis, due to a combination of selection and limits on the production of variation. (*a*) In the depicted trajectories, looking only at the rightmost plane would provide a misleadingly narrow picture of morphospace occupancy. (*b*) A time-extended morphospace illustrating the effects of culturing an SEM (gastruloid) with ECM (Matrigel) supplementation versus without (see Veenvliet *et al*. [29]).

To capture the fact that selection acts throughout the life cycle, morphospace should not be represented solely as the space of possible forms of later developmental stages, when morphogenesis is largely finished and further development is largely limited to the growth of established tissues and organs (see figure 2). Instead, it should be extended in the time dimension, so as to include developmental trajectories that are only viable up until stages prior to completion of morphogenesis. SEMs exhibit truncated trajectories of this sort. Because selection acts throughout development, a time-extended morphospace does not represent selection-free patterns of development.

### 2.3. Developmental constraints or internal selection?

Alberch’s advocacy for the evolutionary importance of ‘internal constraints’—better known as ‘developmental constraints’—points to a longstanding debate over adaptationism and the causes of morphological stasis. Internalists or structuralists have argued that the (hypothetical) sparse filling of morphospace is due largely to developmental constraints on the production of phenotypic variation [14–17,36]. Selection, they argue, plays a more minor role as a ‘filter’ or ‘sieve’ of the variations that are possible to produce developmentally.

What is a developmental constraint? The concept can be divided into mechanistic versus variational meanings (see Glossary). In 1985, a standard variational definition was provided in a paper authored by leading evolutionary and developmental biologists, including Alberch [37]. There, a developmental constraint is defined as ‘a bias on the production of variant phenotypes or a limitation on phenotypic variability caused by the structure, character, composition, or dynamics of the developmental system’ [37, p. 266] (cf. [19]). A conceptual difficulty with this definition is that in order to establish the effect of a ‘bias’ on some process—in this case the production of variant phenotypes—we need a null model or expectation of what that process would look like in the absence of the biasing factor. However, it is unclear how we should picture the production of variant phenotypes as unbiased or unconstrained by development [37]. At least in multicellular organisms, development just is how variant phenotypes are produced. These cannot be compared as independent processes. It is not clear, for example, what evidence could establish what tetrapod limb development would look like unconstrained by actual limb outgrowth and patterning mechanisms. This kind of difficulty has contributed to the avoidance by some more recent authors of the variational notion of developmental constraints in favour of more neutral concepts like genotype–phenotype mapping, variational properties or evolvability (e.g. [38–42]; for further discussion of developmental constraints, see [10,43,44]).

In practice, the null model of the generation of phenotypic variation presupposed in the above definition [37] was an idealized morphospace in which the dimensions are combinatorial and geometric possibilities for a given character and in which variations along any dimension are expected to arise with roughly equal frequency [4,20,45]. Variational developmental constraints could then be detected by deviations from the expectation of equal frequencies for any variant in this morphospace [4,20,46]. Salazar-Ciudad [39] refers to this background expectation as the ‘isotropic expectation’. It loosely corresponded to then-prevailing assumptions among evolutionary biologists that selection is almost entirely responsible for limits on phenotypic variation (e.g. [47,48]; see figure 1). The primary evidence for this view was the finding from artificial selection and breeding experiments that very many arbitrary quantitative traits can rapidly change in response to selection, and thus their normal values must be maintained by stabilizing selection [49–53]. These findings support the hypothesis that much more variation in quantitative traits can be generated than what we observe in natural populations. However, they do not support the isotropic expectation that variation along any dimension is equally likely. Such assumptions are rarely stated explicitly, and so it is not clear how many evolutionary biologists adhered to the isotropic expectation [39].

The idealized null model of morphospace also depended on the idea that certain phenotypes are *genetically* possible but are unlikely or impossible due to the nature of the developmental process that uses those genes. Indirect evidence for this proposal lies in the recognition that all possible genetic changes are continually occurring (insertions, deletions, inversions, etc.), that variation is largely unconstrained at the genetic level and that constraints thus arise from the developmental processes that are affected by genetic changes. This reasoning is valid, but it does not support the existence of a *genetically* unconstrained space of *morphological* variation. Morphological phenotypes are typically only generated through a combination of genetic and non-genetic factors, such as metabolism, biochemistry, cell- and tissue-scale mechanics, mechanochemical feedback, tissue geometry and environmental cues [4–7,54,55]. A hypothetical statement that, for example, a 13-fingered squamate autopod is prohibited by developmental constraints, but is possible at the genetic level, is conceptually unclear. Genes without development do not make an autopod. Altering developmental factors such as tissue tension, cell behaviour and proliferation rate may lead to different morphologies, but this usually has to be established through experiments (which could be conducted with SEMs) rather than being evident *a priori*. Such experiments can reveal the different phenotypic effects of different mechanisms, but not the effects of developmental constraints on otherwise unbiased variation.

**Figure 3.**
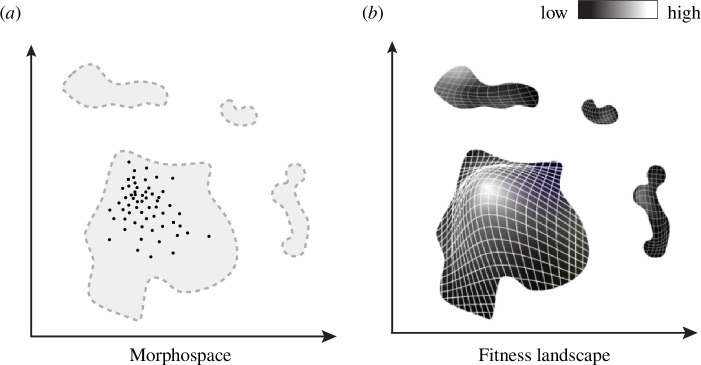
A fitness landscape (*b*) overlaid on accessible regions of morphospace (*a*) captures the fact that selection only occurs within the bounds of phenotypes that are possible to develop. Extending in the time dimension (see figure 2), one can picture fitness landscapes for each successive developmental stage (cf. Oster & Alberch [13]).

One possibility of framing the required decoupling of genotype and phenotype is to note that different genotypes can lead to the same phenotype (genetic *robustness*) and the same genotype to different phenotypes (*plasticity*). While true, this does not justify the idea that a given morphological phenotype is genetically possible *independently* of developmental factors. By default, we should think of possible morphologies as determined by the *entire* suite of generative processes, including genetic and developmental variation acting together, rather than thinking of development as constraining an independently existing field of genetic potential for possible morphologies (see figure 3).

A further difficulty with the concept of developmental constraints deployed by Alberch [4,20] lies in its use as a contrast to natural selection. The idea that development must be the source of constraints on morphological variation because genetic change is constant and less constrained can be understood in the following way. There are developmental constraints when genetic variations arise that disrupt existing developmental processes and so cannot give rise to viable phenotypes. However, this process is just internal stabilizing selection (see [56–58]). Rather than being a limit on the production of variants available to selection, differential viability due to development *is* a type of selection. As Reeve & Sherman [59] pointed out, many of the examples of developmental constraints deployed by critics of adaptationism are features maintained by selection. They redefine developmental constraints from an ‘adaptationist’ perspective as follows: ‘Phenotypes are developmentally constrained when the alternative phenotypes have lower fitnesses because they depend upon developmental processes that would seriously disrupt the original developmental program, thereby reducing viability’ [59, pp. 21–22]. As a general definition of variational developmental constraints, however, this is too narrow. The fact that many (perhaps most) variational constraints arise from internal selection does not preclude the existence of developmental limits on which phenotypic variations can be produced. Yet it is critical to recognize that these are very different kinds of ‘constraints’ on variation: selection is a causal process in which variants that actually arise are ‘constrained’ (sorted), whereas variational developmental constraints are limits on the possibilities imaginable by human scientists that are biologically realizable. The latter are inherently more difficult to establish empirically.

Taking all of the above together, internalist and externalist views of morphospace occupancy can be integrated in the following way. The forms that are developmentally accessible for a given clade define the region(s) of possible generation of variation (figure 3*a*). What lies outside of this bounded space are forms that may be conceivable, or formally ‘possible’ in the sense of corresponding to values of the parameters that we use to define the space. But these hypothetical forms are not biologically possible *given* developmental constraints and phylogenetic initial conditions. Since selection can only act on forms that are actually generated, selection occurs within the bounds of the generative space of variation, on actual variants. Many, but not all, apparent variational developmental constraints may be explainable in terms of internal selection. A fitness landscape [60,61] can accordingly be overlaid on the generative space (figure 3*b*). Experiments on SEMs can modify mechanistic developmental constraints (see below) and thereby change variational constraints on which phenotypic variations are accessible. This is how SEMs can ‘explore’ morphospace, given that SEMs have zero reproductive fitness and so cannot evolve. Developmental processes with different variational properties can be compared with each other, but no experiment can reveal what the generation of phenotypic variants unbiased by development would be.

### 2.4. Pathology provides insight into normal development

Returning to the description of evolution as a two-part process comprising the (i) generation of variants and (ii) sorting of variants, it is clear that development and selection are different parts of the same integrated process. Although we can ask which aspect contributes more causal specificity to a given evolutionary trajectory, both (i) and (ii) are always necessarily involved in evolution. They are not independent causal hypotheses in the same way as, for example, smoking versus air pollution as putative causes of lung cancer. The generation of genotypic and phenotypic variants is part of what it means for evolution to occur, whereas being caused by smoking is not constitutive of lung cancer. Monstrosities and ‘internal constraints’ are not necessarily indicative of what selection can or cannot do. Rather than providing direct insight into evolution, we suggest, developmental monstrosities are interesting for a more basic and general reason: pathological states can provide unique insight into the hidden causal structure of normal states [62,63]. Sigmund Freud had an intuitive metaphor for understanding how mental illness could reveal the structure of ‘normal’ or healthy psychological development:

Pathology […] can draw our attention to normal conditions which would otherwise have escaped us. Where it points to a breach or a rent, there may normally be an articulation present. If we throw a crystal to the floor, it breaks; but not into haphazard pieces. It comes apart along its lines of cleavage into fragments whose boundaries, though they were invisible, were predetermined by the crystal’s structure. [64, p. 4667]

More recent biological work has pursued a similar line of thought. Uri Alon [65] suggests that common human pathologies are orderly enough that they are classifiable into a ‘periodic table’ of disease for different cell types. He theorizes that this order can be explained by physiological circuits that evolved to be robust for certain functions, but that leave them vulnerable to stereotyped and predictable ways of breaking down. Pathology can thus indirectly reveal how the system has been put together.

We suggest that SEMs are tools of discovery for the same reason. They are, compared to Alberch’s naturally occurring monsters, much further removed from the history of selection in the source lineage. They are human-guided developmental systems containing a subset of the components of normal development. This effectively provides a large-scale perturbation—a breaking of the crystal—that can elucidate which components are necessary for reaching certain developmental stages and/or developing certain features *in vivo* such as patterning, tissue architecture and composition. They also allow the detection of viability selection in a way that is practically impossible to observe *in vivo*. For these reasons, the monstrosities observable in SEMs promise to be more revelatory about the internal structure of development than naturally occurring monsters. The next sections describe in more detail the main developmental insights that can be derived from current embryo models.

## 3. Modelling development in a dish: stem-cell-based embryo models

The last decade has seen a sharp rise of SEMs, culminating in these being proclaimed Method of the Year 2023 [66]. SEMs are generated by coaxing PSCs into forming aggregates of a defined size that, when subjected to a specific regime of culture conditions, can *self-organiz*e into structures that model (aspects of) the natural embryo: spatial organization of gene expression domains, differentiation trajectories, morphogenetic processes, etc. By now, a myriad of SEMs exist, spanning pre-, peri- and post-implantation stages up to early organogenesis. Such structures have been generated using mouse and, more recently, human as well as non-human primate stem cells. These include blastoids (which model the blastocyst stage), ETS (embryonic and trophoblast stem cell SEMs) and ETX (embryonic, trophoblast, extra-embryonic endoderm stem cell SEMs), which model peri- and early post-implantation stages, and gastruloids (which model the outcome of gastrulation up to early organogenesis). For detailed accounts on the properties of these models and their generation, we refer to many excellent and extensive recent reviews [67–71]. Here, we focus on the unique characteristics of SEMs that render them suitable to test concepts discussed in the previous section.

### 3.1. Stem-cell-based embryo models are easy to access, track, manipulate and scale

Compared to the natural embryo, SEMs come with multiple experimental advantages. First, whereas the paucity of samples is often a rate-limiting factor for experimentation on embryos, SEMs can be generated in large numbers (usually dozens to thousands per experiment). Second, culturing SEMs in a Petri dish allows easy monitoring and manipulation of the samples, enabling longitudinal analysis of developmental processes as they unfold (traceability). This includes developmental landmarks such as implantation, gastrulation and (early) organogenesis, which are usually hidden from sight owing to the inaccessible intrauterine environment (at least in mammals). Third, the greater manipulability allows for precise control of the parameter space (comprising genetic, biochemical, mechanical and geometrical cues) [5,7]. This facilitates the systematic and quantitative exploration of the effects of these inputs on developmental trajectories and phenotypic outcomes. Notably, all of these advantages become even more prominent in the case of human development, where SEMs offer an ethical and legal alternative to studying the natural embryo [72]. Altogether, SEMs offer multiple unique opportunities for scientific discovery, in particular in the context of the developmental morphospace:

— The generation of large numbers of SEMs from PSCs with the same genetic background enables the observation and quantification of natural variability in phenotypes emerging due to stochastic processes.
— Systematic manipulation of the SEM micro-environment allows the exploration of how environmental factors affect developmental trajectories and phenotypic diversity.
— The high-throughput nature facilitates genetic screens, providing insights into genotype–phenotype relationships.
— The combination of scalability and traceability permits the systematic and quantitative mapping of developmental trajectories (e.g. through backtracking of live imaging data), enabling the acquisition of time-extended morphospaces.
— SEMs can potentially be generated from PSCs from many different species, potentially paving the way for insights on the mechanisms of morphological diversification during evolution.
— Finally, as compared to the natural embryo, SEMs navigate a much larger region of their developmental morphospace, manifesting as phenotypic variation. While such variation represents a nuisance for applications that require high reproducibility (e.g. drug toxicity assays and genetic screens), it offers an opportunity to study how the embryo is shaped by internal versus external constraints (in the mechanistic sense of ‘constraints’; see Glossary).

In the rest of this section, we will discuss the emerging body of knowledge on putative ‘drivers’ of SEM phenotypic variation.

### 3.2. The known: effect of controllable variables

SEMs begin as highly controlled aggregates of PSCs and require numerous external inputs to create a micro-environment that attempts to mimic (aspects of) *in vivo* developmental conditions. Although the systematic study and optimization of controllable variables are more often the exception than the norm [73], a growing body of phenomenological evidence has pinpointed controllable variables that influence phenotypic outcomes; for the in-depth and explicit presentation of these controllables, we refer to comprehensive reviews [7,71,73–75]. Here, we discuss some prominent examples of such variables, which can roughly be grouped into pre-, peri- and post-aggregation variables.

#### 3.2.1. Controllable pre- and peri-aggregation variables

Firstly, both the *genetic background* and the *pluripotency state* of PSCs have been linked to divergent developmental outcomes in various SEMs. Genetically distinct embryonic stem cell lines in mouse (mESC) and human (hESC) differ in their ability to successfully and reproducibly generate SEMs, affecting their cellular composition, shape and patterning under otherwise identical conditions [76–81] (discussed in Veenvliet *et al*. [7]).

Secondly, the *starting number* of PSCs (*N_0_*) ‘seeded’ to form a single SEM appears critical for successful and reproducible formation. For successful gastruloid formation, robust morphogenesis appears to occur over roughly one order of magnitude of *N_0_* (100–1000 cells), with scaling of anteroposterior (AP) gene expression domains. However, this scaling breaks down for any *N_0_* outside of that range [82,83]. Variations that result from *N_0_* changes are also particularly relevant for SEMs that require co-culture of different cell types, where starting cell ratios are crucial for (reproducibility of) SEM phenotypic outcome [84]. Finally, both relative positioning and geometric constraints can influence phenotypic robustness. In 2D micropatterned PSC cultures, geometric confinement can guide the asymmetric patterning of Brachyury [85]. In three-dimensional (3D) trunk organoids, such predictable symmetry breaking can be achieved by the defined positioning of epithelial cysts [86].

#### 3.2.2. Controllable post-aggregation variables

The *composition of the culture media*, with a prominent role for supplemented recombinant proteins (e.g. WNT (wingless-related integration site) and FGF (fibroblast growth factor)) and/or small molecules activating or inhibiting signalling pathways, has a profound effect on protocol outcome and efficiency. For example, careful optimization of culture medium composition drastically improved the efficiency and fidelity of human blastoids [87]. Similarly, while gastruloids break symmetry and *elongate* both with and without FGF and activin, their final cell type compositions differ depending on the presence or absence of these factors [29,88–90]. Other examples are transient supplementation of retinoic acid, which can both increase cell type diversity [91] and stabilize somite formation [92] in human SEMs of the embryonic trunk, and embryo-inspired timely BMP (bone morphogenetic protein) and nodal inhibition, which induces a notochord-like structure in gastruloids [93].

*Mechanical or* mechanochemical *cues* also affect SEM differentiation outcomes. A dramatic example is the induction of coordinated formation of somites and neural tube in gastruloids by precisely timed supplementation with an ECM compound (Matrigel), effectively unlocking embryo-like morphogenesis [29,91,94] (see figure 2*b*). In the context of SEMs of earlier developmental stages, ECM supplementation also appears critical to induce embryo-like morphogenetic processes such as lumenogenesis and cavity formation [95,96].

Finally, *metabolic inputs* have been linked to phenotypic outcomes in multiple SEMs. Culture of gastruloids under physiological oxygen tension (instead of atmospheric) circumvents the need of exogenously provided WNT to trigger symmetry breaking, axial elongation and AP axis formation. Moreover, such physiologically realistic hypoxia increases the reproducibility of gut primordium formation [97]. Such a putative canalizing role of physiological hypoxia extends to other SEMs, as well as *ex utero* culture of natural embryos [98–100]. Other recent work demonstrated that the metabolic state of gastruloids and trunk-like structures influences their morphogenesis and germ layer composition through the regulation of morphogen signalling, with glucose metabolism regulating endodermal and mesodermal representation [101–103].

### 3.3. The unknown: unexplained variation

Even under identical user-defined culture conditions, considerable phenotypic variation in SEMs is observed [7,102,104]. Effectively, this results in the limited efficiency of various SEMs to model their target structure (the natural embryo, or aspects thereof) with high fidelity. Interestingly, there appears to be a trade-off between the ability to achieve embryo-like complexity and formation efficiency, especially for SEMs recapitulating later embryonic stages (early organogenesis). For example, blastoids (embryo-like morphology, early stage) and conventional gastruloids (less embryo-like morphology, later stage) are highly reproducible (70–95% efficiency) [29,84,87,88,104–106]. In contrast, highly complex models aimed at modelling the full conceptus typically show low efficiencies (usually in the 0.1–2% range) [79,107,108]. This trade-off can be viewed as a consequence of internal selection, where greater developmental complexity and/or longer ontogenies allow more opportunities for the developmental variability in SEMs to be disruptive. Notably, even for systems where reported efficiencies are high, the granularity of analysis could mask other forms of variation. For example, even in robustly elongating gastruloids, there is significant heterogeneity in lineage composition and considerable heterochrony [102,104].

Such variation not only is observed in terms of phenotypic outcome but also extends to the developmental paths taken by SEMs. Compared to the natural embryo, the route taken by SEMs can be accelerated or decelerated, and both state divergence and convergence compared to natural embryos have been observed (discussed in [7,109,110]). This includes the remarkable capacity to skip *in vivo* developmental hallmarks: SEMs modelling the day 14 human conceptus do not pass through the blastocyst stage when the natural embryo would normally implant [79], blastoids skip cleavage stages [84,105,107] and gastruloids model the outcome of gastrulation while skipping both stages (at least morphologically) [69,76,88,89,106,111].

Such SEM disparities compared to natural embryogenesis are usually attributed to missing *in vivo* developmental constraints, indicating that an embryo cannot be reduced to an aggregate of PSCs but also includes its environmental context. Indeed, mechanisms that shape and pattern the early embryo include both ‘internal’ ontogenic (e.g. gene expression and cell–cell communication) as well as ‘external’ ontogenic mechanisms (e.g. biochemical inputs from extra-embryonic tissues, nutrients and metabolites, *boundary conditions*). While rational model design, often inspired by known *in vivo* design principles, might therefore increase SEM reproducibility, robustness and/or fidelity (as discussed above), it is unlikely that the full regime of *in vivo* constraints can be recapitulated in a dish. Notably, this is also undesirable, for when a model becomes as complex as its target, it might not be as useful, as it is now just as difficult to understand as its original target [112,113].

From the perspective of Alberch, who suggested that developmental morphospace is highly ordered even in the absence of selection, SEMs are interesting because they allow the expansion of accessible morphospace. They offer the opportunity to explore patterns and limits of variation, as well as the robustness of many phenomena despite increased phenotypic variation (compared to the embryo). Gastruloids are particularly pertinent as an example of the robustness found in SEMs, where the recognizable gastruloid configuration and elongation concomitantly with AP patterning [88,111] is highly robust despite differences in protocols, as well as species differences from which PSCs originate [109,114]. This suggests that, at least for some features and processes, SEMs possess internal mechanisms that can give rise to certain aspects of reproducible form even when external constraints are removed. A prominent example is the gastruloids and zebrafish blastoderm explants that break symmetry and establish a primary body axis in the absence of extra-embryonic tissues, traditionally believed to be pivotal for this morphogenetic event [88,106,111,115,116]. Notably, similar morphogenetic outcomes do not necessarily imply that the molecular mechanisms or cellular behaviours are retained since a wide variety of these can converge on similar physical principles [117].

Altogether, the less constrained nature of SEM development in combination with easy traceability and scalability allows us to extend the boundaries of actual diversity and facilitates the study of ‘pure form’ [4] by lifting mechanistic and/or variational constraints under minimal (external) selection [20]. With this in mind, what we set out to propose in the next section is a programme to leverage the ‘monstrosity’ of SEMs to uncover the organizational and ontogenic rules of the developmental constraints that shape early embryos.

## 4. The relevance of stem-cell-based embryo models for an analysis of developmental ‘monstrosities’

Even though a teratological science of ‘pure form’ as envisaged by Alberch may be out of reach, a careful study of developmental ‘monstrosities’ nonetheless has the potential to provide insights on form. However, monsters are rare and often non-viable, inhabiting parts of the developmental morphospace that are eliminated over time, and therefore difficult to observe in the natural world (figure 1*a*). At the same time, it remains unclear what part of the broader morphospace is accessed during normal development. This is especially true for species (such as mammals) where embryogenesis is more regulative than deterministic, with considerable plasticity and canalization enabling robust phenotypic outcomes in the face of environmental disruptions.

In this section, we set out to show how SEMs represent a powerful platform for the experimental study of developmental anomalies (monsters) and hence could enable the discovery of ‘hidden’ rules of robust natural development.

### 4.1. The logic of monsters

Developmental anomalies *in vivo* are under selection, preventing many teratologies from being born in a viable state as a result of not passing developmental checkpoints (e.g. implantation or gastrulation). Even though SEMs might not be entirely freed from viability selection (as discussed in §2), the selection pressures are different. This is likely what allows them to navigate a broader morphospace with a distribution of developmental trajectories that is more variable than natural embryos. Hence, by revealing otherwise hidden parts of the developmental morphospace and with large sample sizes, SEMs are more potent monsters for study compared to natural or even experimentally induced teratologies, such as the ‘hopeful monsters’ created by forward genetic screens [118].

Increased efforts to systematically characterize SEM variability and how it is influenced by controllable variables have started to shed light on putative principles at play during aberrant development, i.e. the logic of monsters. Let s illustrate this with some prominent examples.

A first example is the human extra-embryoid (hEE), an SEM of human peri-gastrulation events, with the key removal of a developmental constraint that the *in vivo* embryo depends upon: trophoblast derivatives (extra-embryonic tissue fated to form the placenta) [81]. Extensive phenotypic characterization revealed that the structures obtained in a single experiment were not continuously distributed but instead fell into three discrete patterns: embryonic-only structures, extra-embryonic only or structures where an internal epithelial cyst is surrounded by extra-embryonic cells, with the latter organization most reminiscent of (albeit still distant from) the natural embryo. Further analyses revealed that the self-organizing capacity of these latter structures (deemed ‘true’ hEEs) relied on the presence of hypoblast-like cells.

A second example is the thorough characterization of endoderm morphotypes in mouse gastruloids [119]. Quantitative morphometric analysis revealed that definitive endoderm develops into distinct morphotypes in gastruloids, and predictive modelling using live imaging data uncovered variables underlying morphotype choice. These variables could be tuned to lower variation and steer the system towards a more embryo-like gut.

A final example comes from the detailed molecular and morphological characterization of hundreds of individual gastruloids and TLS [102]. This demonstrated that structures that lack *in vivo*-like coordinated formation of a neural tube and somites are either neurally biased with overproduction of disorganized neural tissue, or formed ‘inside-out’, with somitic tissue enveloped by neural tissue. Integration of morphodynamics with molecular signatures powered by predictive modelling suggested that divergent metabolic activity underlay end-state molecular and morphological variation. Indeed, metabolic interventions could partially correct neural bias and aberrant morphology.

These examples show that systematic mapping of the SEM morphospace in combination with detailed, time-resolved analysis of their formation can uncover an underlying logic that reveals design principles ensuring the robust acquisition of shape and patterning in the natural embryo. How do we start constructing exhaustive SEM morphospaces? As the examples given above and other studies show, a powerful approach is high-content imaging, followed by feature selection and clustering [120–124]. An important notion is that the observed orderliness might depend on the parameters plotted on the morphospace axes, which are often guided by the experimental question at hand. Hence, a powerful approach to minimize the contingencies of experimental design is to quantify a larger set of geometrical parameters and apply dimensionality reduction on these features [120,122–124] to capture the main variation in the data. While the exact observed distribution in the developmental morphospace might depend on the experimental model and/or the parameters used, the applicability of quantitative approaches in combination with high sample sizes make SEMs a powerful experimental system to assess how the absence of specific constraints imposed by the *in vivo* environment results in a broader range of morphologies.

### 4.2. Taming monsters by reintroducing constraints

**Figure 4.**
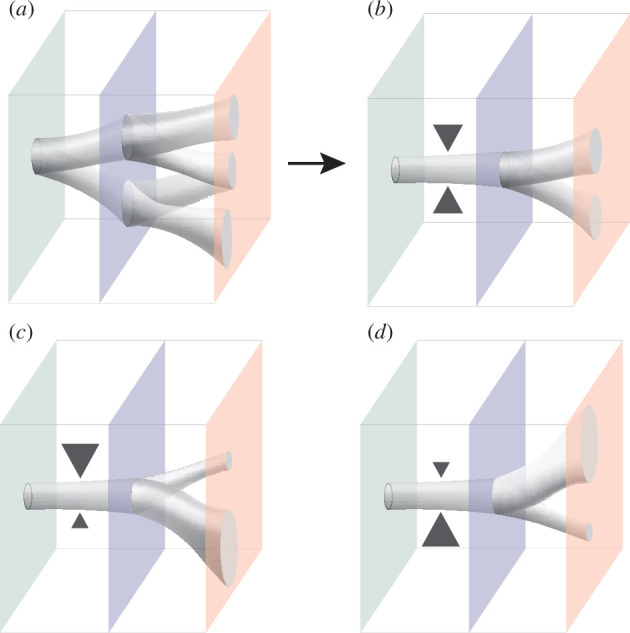
Re-introducing constraints can change the accessible morphospace. (*a,b*) Adding constraints (represented by the triangles) can reduce the variability of developmental trajectories. (*c,d*) Re-introduced (combinations of) constraints can also alter the probability distribution of developmental trajectories (see text for details).

As mentioned in §3, the use of SEMs enables the specific re-introduction of various constraints known to act on natural embryo development. These constraints can take different forms. *Mechanochemical* constraints include extra-embryonic tissues, which can serve as morphogen signalling centres and are a rich source of ECM, which provides physical scaffolding, mediates biochemical signalling and imposes boundary conditions. *Environmental* constraints include, for example, nutrient and oxygen availability. *Geometrical* constraints include, for example, size and shape constraints imposed by the maternal environment. Excitingly, the accessible nature of SEMs enables the precise re-introduction of these constraints, even combinatorially and/or with spatiotemporal control. This approach allows researchers to study how different parameters shape the developmental morphospace of an SEM and can provide insights into how specific *in vivo* constraints canalize development and thereby confer robustness (figure 4).

While, to the best of our knowledge, no study has yet systematically compared the accessible morphospace in SEMs with and without re-introduced constraints, there are various examples of how specific ‘external’ constraints can canalize SEM morphogenesis by reducing variation, steering the SEM towards regions of the morphospace occupied by the natural embryo. For example, as noted earlier, re-introducing ECM into the gastruloid culture unlocks coordinated morphogenesis of a neural tube and somites, resulting in structures that not only mimic natural embryo trunk patterning [29] but also architecture. Adding extra-embryonic endoderm leads to the formation of anterior neural epithelia—a phenotype not observed in traditional gastruloid protocols [125]. In the context of *in vitro* gut primordium development, which displays large variability in gastruloids [29,126], the re-introduction of the *in vivo* constraint of physiological hypoxia greatly enhances formation efficiency [97].

In addition to the re-introduction of known *in vivo* constraints, systematic mapping of SEM developmental morphospace can facilitate the reverse engineering of embryo design principles by pinpointing the key factors driving morphotype variability. These factors can then be tuned, followed by a re-evaluation of the morphospace. The endoderm morphotype example from §4.1 is an excellent example. Upon mapping the morphospace, predictive modelling uncovered that coordination between the stage of endoderm progression and gastruloid elongation is key in morphotype choice. Interventions designed to control this coordination reduced variability and steered morphotype choice, resulting in gastruloids forming an *in vivo*-like gut-tube more reproducibly [119]. These examples show how the re-introduction of constraints can reduce the region of the morphospace accessed by SEMs (figure 4*b*) or alter the distribution by affecting state transition probabilities (figure 4*c*,*d*).

An interesting additional possibility of SEMs is that they allow us to impose artificial constraints that do not exist *in vivo*. The chemical coupling of trunk organoids is a prominent example. A particular spatial arrangement (identified by machine learning on high-content imaging screens) of these SEMs synchronizes their oscillation dynamics, which restricts the degrees of freedom of their axial patterning system. This reduces the system’s entropy, thereby achieving robust and predictable symmetry breaking and AP axis formation [86,127]. In this way, bestowing a novel variational constraint condensed the region of morphospace occupied by the SEM.

The tractability of SEMs also facilitates the investigation of the feedback interactions that give rise to self-organization in morphogenesis. In gastruloids, for example, the formation of a heart tube can be induced by supplementing with known cardiogenic factors [128], but efficiency appears to be ESC genetic background dependent and is associated with the *in vitro* formation of a gut primordium, which provides mechanochemical cues *in vivo* [129,130]. In mouse blastoids generated by co-aggregation of trophoblast and embryonic stem cells, ESC-derived signals drive trophectoderm morphogenesis [84], and reciprocal inductions between the epiblast and extra-embryonic endoderm support progression into post-implantation stages [131]. In both cases, targeted manipulations of the individual components (lineages) affect morphogenesis [84] or developmental progression [131]. Such findings suggest that the versatility of SEMs can be leveraged to dissect how combinatorial inputs affect the navigation of the developmental morphospace.

It is important to consider a particular nature of constraint that remains to be explored in SEMs: the *historical* one. ESCs are derived from the blastocyst, meaning that the starting material of SEMs has been subjected to prior *in vivo* evolutionary forces, as well as developmental constraints, acting upon the embryo until that stage. This is important, since the mere act of passing through successive developmental stages can inform the trajectory of future embryonic outcomes. For example, events as early as the two-cell stage give rise to imbalanced clonal cell populations in mouse and human embryos [132]. It remains unclear to what extent such historical constraints are carried over to ESCs in culture and affect the developmental morphospace that can be explored by SEMs. This might be even more true for SEMs derived from induced PSCs, since these often carry (epi)genetic signatures of the tissue of origin [133]. In this regard, it is noteworthy that blastoids (which model blastocysts) show, under defined cultured conditions, little morphological variation compared to SEMs of more advanced developmental stages. One might speculate that ‘process memory’ of prior blastocyst formation capacitates ESCs with the potential to model target stages experienced up until their derivation with high fidelity and reproducibility, while for subsequent stages the absence of such historical effects results in wider divergence in the phenotypic space. Nevertheless, passing through previously experienced stages is not obligatory since many SEMs of peri- and post-implantation do not, at least morphologically, pass through the blastocyst developmental stage.

### 4.3. Minimal models for the study of system interactions

While it would be possible and interesting to conduct both forward and reverse genetic screens with SEMs and map the full developmental morphospace accessed through these perturbations, probably their strongest feature is that they allow us to see the effects of large-scale non-genetic perturbations. Alberch noted that ‘…we cannot have a purely genetic theory of morphological evolution. Theory of form has to be based on the global properties of the network of interactions that characterize development’ [4, p. 43]. Specifically, he depicted extensive nonlinear feedback between genetics, biochemistry, geometry and mechanics shaping the embryo, which he referred to as the cyclical (versus hierarchical) view of development. In SEMs, a wide range of possible forms emerges from the same genetic material, cultured under the same conditions, which means that the observed variation must have originated in disruptions of non-genetic information modules. Hence, SEMs provide a unique opportunity to assess the phenotypic consequences of perturbations of ‘properties that emerge from the dynamics of the system and are not encoded in the genome’ [134, pp. 6–7].

Given the burgeoning evidence that development indeed rests upon the interactions and interdependencies of gene-regulatory networks (GRNs) that specify cell fate and cell-regulatory networks (CRNs) [6] that specify multicellular ensemble behaviours, SEMs offer an experimental window into studying these networks in partial isolation, as well as the interactions between them that occur upon experimental manipulation [6,109]. As discussed in Steventon *et al*. [109], both 2D micropatterns and 3D gastruloids constitute models that do not undergo proper morphogenesis. Yet, even in the absence of proper morphogenesis, spatially organized gene expression domains are still established. Within such systems, the introduction of embryo-like boundary conditions can reinstate morphogenesis without prominent changes in GRNs and gene expression patterns underlying cell differentiation [29,89,91,135]. Such facultative coupling of information modules deeply entangled in the context of the natural embryo [5] allows the exploration of the linkage of GRNs and CRNs through targeted and specific disruption of either one [6,7]. Moreover, comparative analysis of SEMs with different degrees of coupling of cell type specification, patterning and morphogenesis might shed light on information transfer from (sub)cellular to organismal scales (developmental emergence), as well as on the inverse flow of information (downward causation, e.g. morphogenesis producing forces driving flows, changing the apposition of signalling and responding tissues, ultimately affecting gene expression) [114]. The accessibility of SEMs for making precise genetic, biochemical and biophysical measurements and manipulations [7], coupled with *in toto* imaging techniques and mathematical modelling approaches [7,136,137], provides an exciting opportunity to probe and perturb how such bi-directional information flow shapes the developmental morphospace.

### 4.4. Making monsters: expanding the developmental morphospace

In a later work, Alberch [134, p. 10] asked: ‘Why are we unable to create qualitatively new forms in the laboratory?’, and concluded that because we are dealing with evolutionarily fixed developmental systems, experimentally induced perturbations can only explore limited potentialities of the systems, being bound to the reiteration of forms already realized during evolution [134]. Even if not entirely freed from selection, SEMs seem to lift this limitation and indeed acquire forms and take developmental paths not observed *in vivo*. Moreover, the tractability of SEMs offers an exciting opportunity to further expand the occupied morphospace through controlled perturbations. For example, a high-throughput phenotypic compound screen in gastruloids vastly increased their end-state morphospace [120]. Interestingly, the resulting structures could be classified into distinct phenotypes, suggesting that even upon large expansion of the gastruloid phenotypic space, some degree of discreteness persists. Another example comes from SEMs of somitogenesis. Hyper-activation of WNT signalling in a mouse SEM of the embryonic trunk (TLS) results in an overproduction of somites at the expense of neural tube formation, a phenotype not previously observed *in vivo* [29]. A similar phenotype was later reported in a human SEM of somitogenesis [138]. Strikingly, in both cases, the *in vitro* somites acquire a different shape compared to *in vivo* somites: they appear more spherical and are arranged like a ‘bunch of grapes’ [29,138]. Interestingly, such increased rounding and separation compared to the natural embryo is predicted in a simulation-based phase diagram of zebrafish somite formation under a parameter regime where heterotypic tension (at the somite boundary) is at least twofold higher than lateral boundary tension [139], further suggesting that *in vivo* boundary conditions determine somite shape.

Experimental approaches aimed at purposefully increasing ‘monstrosity’ might also be useful to identify bifurcation points and critical decision points where small changes in parameter values describing a developmental process alter the number or probability of phenotypic outcomes (attractor states), and bifurcation boundaries, where equally small perturbations of the parameter space can cause the system to transition from one state into the other [13,140,141].

Finally, detailed mapping of both normal and monstrous developmental morphospaces, together with advancements in our understanding of the ‘logic of monsters’, could allow us to rationally design experimental approaches to engineer SEMs that could fill the voids (i.e. the empty parts) of a given morphospace [121]. As proposed before in the context of organoids, such artificial designs might even pave the way towards novel functionalities [121,142].

### 4.5. Convergence in developmental morphospaces

As briefly discussed in previous sections, extending morphospace in the time dimension can shed light on the developmental paths (or modes) that can be taken to robustly reach the same (or similar) structure. Such convergence of paths was recently described in a study employing a variety of organoid systems, including epiblast organoids, to identify generic rules of lumen formation. Although there were differences in the details, all organoid systems tested showed biphasic development, with initial divergence in organoid shape and topology converging on the same end state [143]. Such convergence can be extended to modalities beyond morphology. For example, recent work used molecular recording to reconstruct lineage trajectories and progenitor fields, which demonstrated that highly divergent progenitor paths can converge on robust developmental outcomes [144]. A final example comes from dissociation–reaggregation experiments in the sea anemone *Nematostella vectensis*, with aggregates taking an alternative developmental trajectory compared to *in vivo*, but converging on the same *bauplan* [145].

In addition to such modes of divergence–convergence, there might also be cases in which the release of *in vivo* constraints condenses the accessible morphospace compared to the natural embryo. This can, for example, become apparent when multi-species comparisons are included in developmental morphospaces. While previous examples illustrate that relaxing developmental constraints can broaden the accessible morphospace for a single species, lifting the same (set of) constraints could potentially condense the developmental morphospace when considering interspecies comparisons. As previously discussed in Steventon *et al*. [109], the release of *in vivo* constraints, in particular species-specific boundary conditions, is what might underlie the emergence of a common morphology in multiple SEMs prior to the phylotypic stage [109,146].

### 4.6. Limitations of stem-cell-based embryo models

As we have illustrated, a major strength of SEMs is that their minimal, bottom-up nature allows us to decouple developmental processes from the inherent complexity of *in vivo* embryo development, where regulative redundancies and viability constraints inevitably limit the accessible morphospace. It should be stressed, however, that SEMs are merely *models* of mammalian embryos that, like all experimental approaches, are not without limitations. Firstly, the developmental potential of SEMs is limited, and they are unable to complete the reproductive cycle. This limits the potential for studying reproduction-related traits [147] with SEMs. While their lack of reproductive capacity prevents SEMs from evolving in response to selection, we can use them to study internal selection on viability (i.e. differences in the ability to progress to later developmental stages).

Secondly, sole reliance on morphological features as parameters defining a morphospace could obscure instances of different developmental mechanisms converging on similar morphological outcomes. While these phenomena are of fundamental biological interest, they warrant caution about inferring shared mechanisms from similar positioning in morphospace, especially in cases where there is weak linkage between GRNs and CRNs. Moreover, the fact that different developmental mechanisms can lead to the same morphological outcomes further highlights the importance of recording time-extended morphospaces and complementing morphometry with other data modalities that capture the GRNs and CRNs driving morphogenesis.

Thirdly, a critical premise in studying biological variation is the minimization of technical variation, for example, due to batch-to-batch differences [74] in ill-defined medium compositions or inter-user variation in the execution of protocols. To mitigate this, reporting standards are essential [148], and detailed reference morphospaces should be established for each SEM protocol.

Fourthly, to evaluate SEMs in the context of the *in vivo* morphospace, the referent morphospace for SEMs has to be defined by the same parameters we use to describe the space of natural embryos. However, defining SEMs with natural parameters could prevent us from fully capturing the novel (regions of the) morphospace they explore. Focusing on broader process outcomes (e.g. patterning and spatial arrangement of tissues) rather than specific parameters, in combination with the inclusion of developmental trajectories, could circumvent these challenges that come with evaluating SEMs using parameters that are biologically or evolutionarily ingrained in natural systems.

Finally, while SEMs allow us to study processes of interest in isolation (e.g. symmetry breaking without extra-embryonic tissues and somitogenesis in the absence of a neural tube) and can thereby uncover autonomous developmental processes that are otherwise masked or influenced by surrounding tissues *in vivo*, the lack of interactions and feedback mechanisms present in the natural embryo means that, as opposed to ‘natural monsters’, design principles inferred from studying monstrous SEMs are not necessarily consequential for *in vivo* development and viability. Likewise, not all mechanisms conferring robustness *in vivo* will be observable in SEMs, for example, because SEMs might not contain the cell types or environmental buffers that are necessary for rescue and error correction [149].

## 5. Conclusion

‘All happy families are alike; each unhappy family is unhappy in its own way’.—Leo Tolstoy

Multicellular development is staggeringly complex, involving regulatory cascades of thousands of regulatory genes operating in feedback with physical processes across multiple scales. This limits what we can learn about biological form—both actual and possible—by perturbation experiments targeting individual genes. Novel SEM techniques represent a significant advance in this regard because they allow for large-scale manipulation and observation of structural constraints on developmental morphology. We have shown how SEMs are enabling the experimental exploration of morphospace roughly along the lines of the ‘monsters’ discussed by Alberch [4]. We suggested that developmental monstrosities are not as selection-free as Alberch thought, due to the indirect influence of past selection as well as internal viability selection during development. Although this challenges any direct implications of monstrosities for the importance of selection in evolution, it does not make SEMs any less interesting for understanding developmental *morphospace*. SEMs can be used to systematically test the effect of *in vivo* mechanistic constraints and to probe how feedback between GRNs and CRNs can shape developmental morphospaces. Interestingly, SEMs can also model internal viability selection across developmental stages in natural embryos, granting us a window into the possible embryonic forms that fail to make it to later developmental stages.

What should such an ‘Alberchian’ research programme on SEMs look like? Firstly, systematic mapping of the SEM morphospace is needed via detailed quantifications of morphology. Ideally, such analysis should include a comparison with the natural embryo, span multiple levels of organization (cells, tissues, organs and organism) and be extended with the time dimension to assess developmental paths taken and identify instances of convergence, putative bifurcation points and possible dead ends. The resulting high-dimensional feature spaces could be represented as phase diagrams upon dimensionality reduction. Secondly, the data should be leveraged to find the underlying rules and constraints, i.e. the logic of monsters. This can be facilitated by powerful new experimental and analytical frameworks, such as (live) imaging in combination with predictive modelling [102,119], molecular recording [144], data-driven mathematical modelling [143,150], integration of data modalities (e.g. imaging and transcriptomics [102], or combinations thereof [124,142]). Thirdly, newly identified putative regulators and/or known *in vivo* design principles can be re-introduced. Following re-introduction, the SEM morphospace must be re-evaluated and factors that condense the region of the developmental morphospace occupied by SEMs to that occupied by the natural embryo can be interpreted as having ‘developmental constraint’ capabilities. Finally, SEMs can be subjected to perturbations to extend possibilities in morphospace. Do such perturbations make the gaps accessible? Can we identify bifurcation boundaries where small changes in the parameter space make the system jump from one state into another? The overall experimental paradigm, integrating multi-scale, multi-modal four-dimensional measurements, mathematical modelling and perturbations, bears similarities to the ‘cytosystems dynamics’ approach proposed by Yoshiki Sasai in 2013 [136]. While visionary, he did not link this to the ‘logic of monsters’ ideas from Alberch, and only with recent technological progress is such a holistic approach now brought within the realms of feasibility. In this way, the idea of an Alberchian research programme on embryo models illustrates how technical progress enables us to address longstanding biological questions, and how conceptual progress refines the questions we are able to ask.

## Data Availability

This article has no additional data.
